# CircLDLRAD3 inhibits Oral squamous cell carcinoma progression by regulating miR‐558/Smad4/TGF‐β

**DOI:** 10.1111/jcmm.17898

**Published:** 2023-08-10

**Authors:** Xue Zhang, Guang‐Yu Guo, Ru‐Yue Liu, Ting Wu, Zhen‐Hua Wang, Zhong‐Ti Zhang

**Affiliations:** ^1^ The VIP Department School and Hospital of Stomatology, China Medical University Shenyang China; ^2^ Department of Surgical Oncology and General Surgery The First Hospital of China Medical University; Key Laboratory of Precision Diagnosis and Treatment of Gastrointestinal Tumours (China Medical University) Shenyang China; ^3^ Department of Physiology, School of Life Sciences China Medical University Shenyang China

**Keywords:** circLDLRAD3, circular RNA, miR‐558, OSCC, Smad4

## Abstract

Oral squamous cell carcinoma (OSCC) is a malignant neoplasm with high mortality and morbidity. The role of circRNA and its molecular mechanism in OSCC remains largely unknown. The study aims to explore the role of a novel circular RNA (circLDLRAD3) in OSCC and its underlying mechanism. PCR and fluorescence in situ hybridization were used to explore the expression features of circLDLRAD3 in OSCC. The effects of circLDLRAD3 on the behaviour of OSCC were investigated using CCK‐8, colony formation assay, transwell and animal experiments. Bioinformatics analysis along with dual luciferase reporter assay and RIP assay were used to reveal the interaction between circLDLRAD3, miR‐558 and Smad4. It was revealed that circLDLRAD3 exhibited low expression status in OSCC. CircLDLRAD3 inhibits proliferation, migration, and invasion of OSCC cells both in vitro and in vivo. Mechanistically, circLDLRAD3 could bind with miR‐558 to positively regulate its target gene Smad4 expression. Rescue experiments further confirmed both miR‐558 overexpression and Smad4 knockdown could reverse the influence of circLDLRAD3 on OSCC phenotypes. Moreover, circLDLRAD3 regulate the TGF‐β signalling pathways to influence EMT through miR‐558/Smad4 axis. Our study found that circLDLRAD3 is downregulated in OSCC and verified its tumour suppressor function and mechanism in OSCC through sponging miR‐558 to regulate miR‐558/Smad4/TGF‐β axis. The characterization of such regulating network uncovers an important mechanism underlying OSCC progression, which could provide promising targets targeted therapy strategies for OSCC in the future.

## 1 INTRODUCTION

Oral squamous cell carcinoma (OSCC) is a malignant neoplasm originating from keratinocyte affecting the lip and intraoral mucosa, accounting for 90%–95% of malignancies in this anatomical region^1^, ^2^ and is one of the top eight causes of cancer‐related deaths worldwide.^2^ The high mortality and morbidity are mainly attributed to delayed diagnosis of OSCC. A large amount cases of OSCC were diagnosed at an advanced stage, often accompanied with lymph node and distant metastasis.^3^, ^4^ Currently, the 5‐year overall survival rate of patients with OSCC is estimated to be 50%–60%. Therefore, it is urgent to elucidate the molecular mechanisms of development and progression of OSCC pathogenesis.

Non‐coding RNAs (ncRNAs) are a class of RNAs which lack coding functions and have recently been found to regulate a variety of biological processes.^5^, ^6^, ^7^, ^8^ With the development of RNA sequencing and microarray technology, circRNAs have gradually been recognized as important tumour biomarkers and present evident regulatory role in various types of cancers.^7^, ^9^, ^10^, ^11^, ^12^ In addition, with the help of advanced detection technologies, multiple miRNA binding sites on circRNAs were detected, which can play a role in regulating protein expression by binding to each other, and abnormally expressed circRNAs can act as miRNA sponges and participate in cancer‐related signalling pathways, thus affecting the progression of squamous carcinoma in patients.^13^, ^14^ However, the role of circRNA as well as its molecular mechanism in OSCC tissues is largely unknown.

Smad4, a central signalling component of transforming growth factor β (TGF‐β), is a multifunctional cytokine that regulates cell growth and differentiation.^15^, ^16^, ^17^ Smad4 was regarded as an important tumour suppressor in various types of cancers.^18^, ^19^, ^20^, ^21^ The role of Smad4 as a tumour suppressor was initially identified in pancreatic cancer as being absent in pancreatic cancer 4 (DPC4)4,^22^, ^23^, ^24^ and since then loss of Smad4 has been identified in a key driver in skin cancer,^25^ head and neck cancer,^26^ and other cancers.^16^, ^19^ Epithelial deficiency of Smad4 could lead to overexpression of TGF‐β which then was released into the tumour microenvironment to promote squamous carcinoma progression through pro‐inflammatory and immune evasion mechanisms.^19^, ^27^, ^28^ However, mechanisms governing dysregulation of Smad4 in OSCC was unclear. It should be further investigated to gain insight into prognosis and therapeutic approaches to potentially guide future clinical trials and improve the prognosis of patients with OSCC.

Herein, we identified a circRNA (circbase ID: has_circ_0006988), which was markedly downregulated in OSCC. We named this circRNA as circLDLRAD3 because it was derived from the LDLRAD3 gene. A series of functional experiments confirmed that circLDLRAD3 could inhibit OSCC cells malignant phenotypes by targeting miR‐558 to positively regulate Smad4, a downstream target of miR‐558. Our results indicate that circLDLRAD3 plays a crucial role in inhibiting OSCC proliferation and invasion both in vitro and in vivo and also revealed a regulatory mechanism of circLDLRAD3 on Smad4 in OSCC.

## 2 MATERIALS AND METHODS

### 2.1 Patient samples

Our study collected specimens from 69 patients who were diagnosed with OSCC and who received maxillofacial surgery at the Stomatological Hospital Affiliated to China Medical University. The specimens were stored at −80°C for long‐term preservation. Patients were diagnosed with OSCC (the diagnosis was confirmed by two independent professors of pathology). This research was authorized by the ethics committee of School and Hospital of Stomatology, China Medical University and all patients signed informed consent. The relevant clinical information and pathological characteristic data were collected and used for statistical analysis.

### 2.2 Cell culture

The squamous carcinoma cell lines CAL27, SCC9, SCC15 and human normal oral epithelial keratinocytes HOK, purchased from ATCC (American type culture collection) were used in our study. HOK cells were cultured in DMEM high glucose medium containing 10% foetal bovine serum. SCC9 and SCC15 cells were cultured in DMEM/F12 medium containing 10% foetal bovine serum. Since all cells were cultured in a common incubator, antibiotics 100 μL/mL penicillin and streptomycin were added to the medium. All cell lines were cultured in a standard cell incubator at 37°C with 5% CO_2_ and constant humidity. The cell lines were routinely examined for contamination and were free of any mycoplasma contamination.

### 2.3 Quantitative real‐time PCR (qRT‐PCR)

TRIzol (Invitrogen) was used to extract total RNA from both tissues and cultured cells using the manufacturer's protocol. Reverse transcription was performed with the cDNA synthesis kit (TAKARA, Japan) to generate cDNAs. SYBR Green Master Mix (TAKARA) was then used for quantitatively examine the relative expression of RNA. For miRNAs, the cDNA was produced through the miRNA first chain synthesized kit (AKARA). Relative expression levels of circRNA, miRNA and mRNA were calculated using the 2^−ΔΔCt^ method through the normalization to U6 or GAPDH mRNA level, respectively. The primers used were from Sangon Biotech and the primer sequences were listed below: circLDLRAD3‐Forward: GGAGCAGAATGCGTCGGAAG, circLDLRAD3‐Reverse: TCACAAAC ACCTGCCCACTG; GAPDH‐Forward: CAGGAGGCATTGCTGATGAT, GAPDH Reverse: GAAGGCTGGGGCTCATTT; E‐cadherin‐Forward: TGCCCAGAAAATG AAAAAGG, E‐cadherin‐Reverse: GGATGACACAGCGTGAGAGA; N‐cadherin‐Forward: GGCTTCTGGTGAAATCGCAT, N‐cadherin‐Reverse: TCCACCTTAAA ATCTGCAGGC.

### 2.4 Cell transfection

miRNA mimics, miRNA inhibitor and their negative controls were obtained. shRNA specifically targeting circLDLRAD3 and the control shRNA‐NC were synthesized and purchased from Genepharma. Lipofectamine 3000 (Invitrogen) was used to transfect these above vectors into cells as the manufacturer's instructions described.

### 2.5 Lentivirus infection

To make lentivirus, shRNA for circLDLRAD3 and the negative control were cloned into the lentivirus vector pLV‐CMV. The constructs were transfected into HEK‐293 T cells together with pSPAX2and pMD2G. The virus‐containing medium was collected twice (24 h and 48 h after transfection). Lentivirus was concentrated through centrifugation at 25,000*g* for 2 h at 4°C. The virus was resuspended in PBS and then used to infect cancer cells for 12 h. After the cells were infected with virus for 72 h, they were examined under a fluorescence microscope and the cells expressed green fluorescence uniformly, and the cells were cultured with 8 μg/mL of puromycin in maintenance screening. After five consecutive passages, each group of cells was collected, RNA was extracted and RT‐qPCR was performed to detect the expression level of the target gene to verify the efficiency of stable transfer and to determine the success of stable transfer of stable screening cells.

### 2.6 Protein extraction and western blotting

Whole cell lysates were made in KeyGen protein extraction kit (KeyGen) and resolved on 3%–8% or 4%–12% gradient SDS‐PAGE gels as previously described.^29^ The gels were then transferred to PVDF membranes. The PVDF membranes with proteins were blocked with 5% blocking milk in TBST, incubated with the indicated primary antibodies overnight at 4°C, and incubated with HRP‐conjugated secondary antibodies. The proteins were then detected using an ECL kit and visualized in a Tanon 5200SF Imaging System. The antibodies used are listed in the Table S1 (Additional file 1: Table S1).

### 2.7 Dual luciferase assay

The dual luciferase reporter gene of circLDLRAD3 and miR‐558, and synthesize mutant and wild‐type vectors for the target site of miR‐558 in circLDLRAD3 were constructed by Genepharma, labelled as circLDLRAD3‐miR‐558 wild‐type (WT), circLDLRAD3‐miR‐558 mutant (Mut). The plasmids were transfected with Lipofamine 3000 according to the manufacturer's protocol, and the experiments were performed 48 h after transfection. The fluorescence activity was detected by the Dual‐Luciferase reporter assay system from Promega (Promega). Cells were collected and then incubated with lysis buffer shaken for 15 min at room temperature. After centrifuge, the supernatant was collected in a new tube and then added to a 96‐well plate (white double luciferase plate) to run the assay via Infinate M200 PRO microplate reader (Tecan).

### 2.8 Transwell assay

To verify the migration capacity of OSCC cells, a sample of 1.0 × 10^5^ OSCC cells was seeded into the upper chamber of transwell devices with 200 μL DMEM medium. A total of 700 μL of DMEM medium including 10% FBS was added to the lower chamber. After incubating at 37°C for 48 h, the cells in the upper chamber were gently removed. After fixing with pure methanol for 1 min, the insert was stained with haematoxylin for 3 min and then eosin for 30 sec. The average number of migratory cells was counted in 10 randomly chosen visual fields under an inverted microscope (Leica DMI300B).

### 2.9 Colony formation assay

The OSCC cells were transfected with circRNA plasmids or miRNA mimics for 48 h. Thereafter, cells were planted into three 6‐cm cell culture dishes (1000 per dish) and incubated for 12 days. Plates were washed with PBS and stained with Giemsa. The number of colonies with more than 50 μm was counted. The colonies were manually counted by microscope.

### 2.10 RNA immunoprecipitation (RIP) assay

In this study, RIP experiments were performed using the Magna RIP RNA‐binding protein immunoprecipitation kit (Millipore) and the Ago2 antibody (Abcam, #ab32381) following the manufacturer's protocol. The qRT‐PCR was performed to examine expression levels of circRNA and miRNA.

### 2.11 Cutaneous xenograft model

All mice were raised in a specific‐pathogen‐free (SPF) environment. Five‐week‐old female BALB/c nude mice were randomly divided into four groups of five mice each. SCC9 cells stably expressing circLDLRAD3 were injected subcutaneously into the right axilla of mice, respectively. During the experimental period, the tumour diameter was measured every 5 days by callipers after the discovery of obvious masses in mice. Tumour volumes were calculated using the formula V = 1/2 L × W^2^. At the end of experiment, the cutaneous tumours were isolated and photographed.

In tail vein injection experiments, SCC9 cells stably expressing circLDLRAD3 and negative controls were injected into the tail vein of nude mice separately to produce an in vivo lung metastasis model. Mice were executed 30 days after tail vein injection, and lung tissues were extracted to make tissue sections.

All animal experiments complied with the ARRIVE guidelines and were carried out in accordance with the National Institutes of Health guide for the care and use of Laboratory animals (NIH Publications No. 8023, revised 1978) and were approved by the Institutional Animal Care and Use Committee (IACUC) of China Medical University.

### 2.12 Statistical analysis

All experiments were performed in three independent replications. Statistical analysis was performed with SPSS 17.0 and GraphPad Prism 8 statistical software. The results were expressed as mean ± standard deviation (SD). The statistical difference was assessed by Student's *t*‐test (two‐tailed). Multi‐group analyses with more than two groups were performed using anova. Correlations between circLDLRAD3 expression and clinicopathological characteristics were analysed using the *χ* ^2^ test. Correlation between the two variables was determined using Spearman's correlation test. Survival curves were calculated using the Kaplan–Meier method, and significance analysis was performed using the Log‐Rank test. P values were expressed as follows: *p < 0. 05, **p < 0. 01, ***p < 0. 001.

## 3 RESULTS

### 3.1 circLDLRAD3 was identified to be downregulated in human OSCC cell lines as well as cancer tissues

**FIGURE 1:**
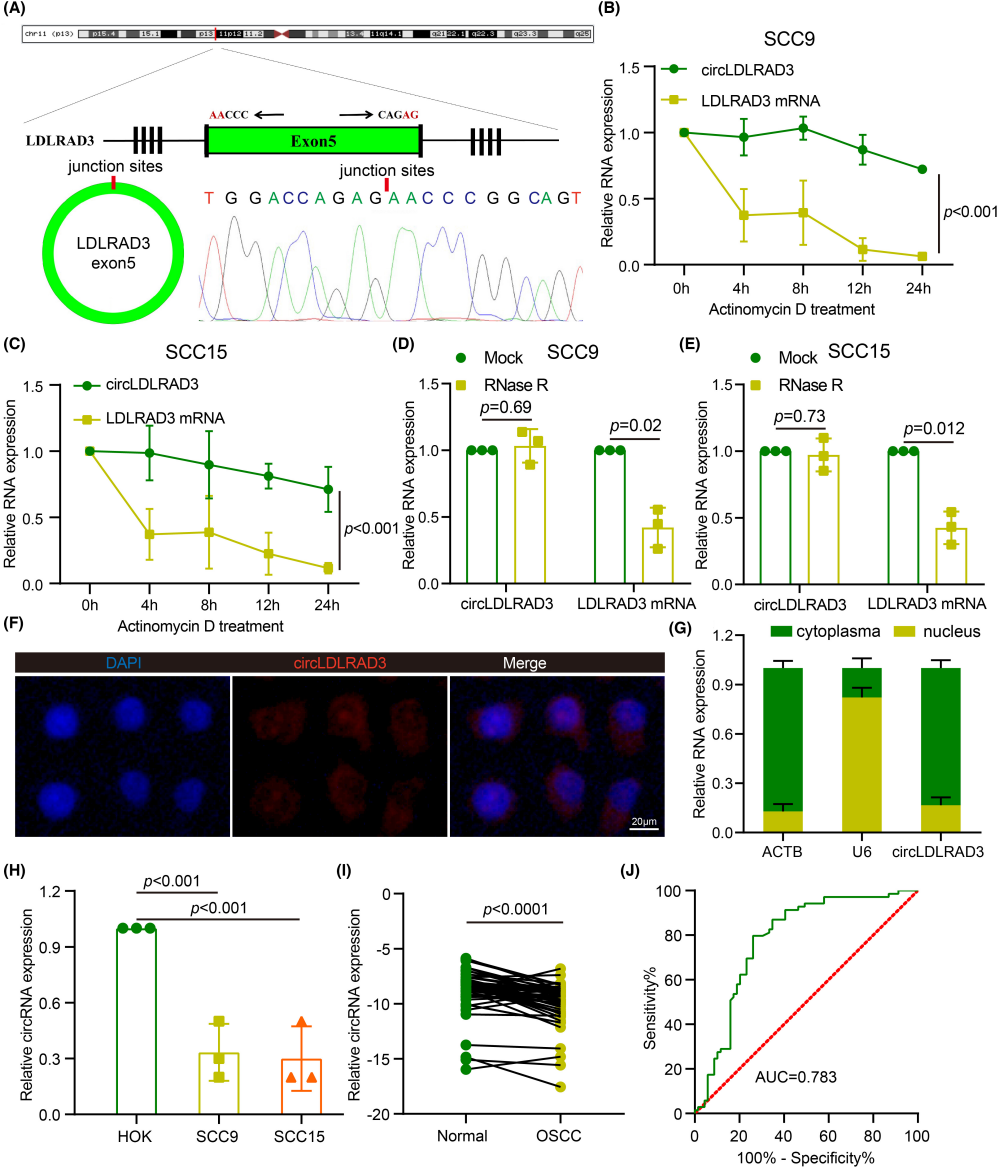
circLDLRAD3 was downregulated in human OSCC. (A) The structure of circLDLRAD3 and the sanger sequencing results for junction site of circLDLRAD3. (B, C) qRT‐PCR to measure expression levels of circLDLRAD3 and linear LDLRAD3 mRNA in SCC9 (B) and SCC15 (C) cells treated with actinomycin D. (D, E) Expression levels of circLDLRAD3 and linear LDLRAD3 mRNA in SCC9 (D) and SCC15 (E) treated with RNase R. (F) Representative FISH images of circLDLRAD3 and DAPI staining. (G) Nuclear/cytoplasmic fractionation assays combined with qRT‐PCR validated the localization of circLDLRAD3 expression. (H) Expression levels of circLDLRAD3 in OSCC cell lines SCC9, SCC15 and normal oral epithelial keratinocytes HOK. (I) Expression levels of circLDLRAD3 in OSCC tissues. (J) ROC analysis of diagnostic efficacy for circLDLRAD3 in distinguishing OSCC and normal tissues. Scar bar = 20 μm; ** *p* < 0.01; ***p < 0.001.

The data from circBank (http://www.circbank.cn/) showed that circLDLRAD3 was derived from the gene LDLRAD3. According to the sequence and junction sites, we designed divergent primers for circLDLRAD3. The qRT‐PCR results and sanger sequencing results further confirmed the specificity of the divergent primers. As the result from Figure 1A showed, circLDLRAD3 is derived from exon 5 of the LDLRAD3 (346 bp) (Figure 1A). As circRNA was reported to be stable than linear RNA, we used actinomycin D to treat OSCC cells for different time‐points to compare the RNA stability difference between circRNA and linear RNA. The results from two OSCC cell lines both confirmed that linear mRNA for LDLRAD3 was easily decayed, while circLDLRAD3 remain at a high level upon actinomycin treatment (Figure 1B,C). Similarly, we further showed that circLDLRAD3 was more resistant to RNase R treatment as compared with the linear LDLRAD3 (Figure 1D,E). Subsequently, the subcellular localization of circLDLRAD3 was explored through fluorescence in situ hybridization (FISH), showing that circLDLRAD3 mainly localized in the cytoplasma (Figure 1F). Further nuclear/cytoplasmic fractionation assays combined with qRT‐PCR validated the localization of circLDLRAD3 expression (Figure 1G).

To probe the expression profile of circLDLRAD3 in OSCC, qRT‐PCR was performed to detect the expression of circLDLRAD3 in OSCC cell lines SCC9, SCC15 as compared with normal oral epithelial keratinocytes HOK. The results showed that circLDLRAD3 was significantly downregulated in these cell lines (Figure 1H). Subsequently, we further detected the expression of circLDLRAD3 in human OSCC tissues, showing circLDLRAD3 expression in OSCC tissues was significantly lower than the paired adjacent tissues (Figure 1I). ROC (Receiver operating characteristic) curve was then utilized to evaluate the diagnostic efficacy of circLDLRAD3 in distinguishing OSCC and normal tissues. The data showed that circLDLRAD3 expression could effectively distinguish OSCC and normal tissues, with an AUC (area under the curve) value of 0.783 (Figure 1J). Next, we assessed the relationship between circLDLRAD3 expression and the patients' clinicopathological characteristics. The results of analysis revealed that the expression of circLDLRAD3 was negatively associated with lymphatic metastasis (*p* < 0.05) and TNM stages (*p* < 0.05), but showed no significant association of gender, T stage, smoking, drinking etc (Table 1). In brief, these data showed that circLDLRAD3 exhibited low expression status in OSCC and may function as a tumour suppressor.

**TABLE 1:** The relationship between circLDLRAD3 expression and clinicopathological parameters.

Clinicopathological parameters	Cases (*n* = 69)	CircLDLRAD3 expression		*p* Value
		Low (%)	High (%)	
Gender				0.778
Male	54	23(42.59)	31(57.41)	
Female	15	7(46.67)	8 (53.33)	
Age				0.863
≤60	23	13(56.52)	10(43.48)	
>60	46	27(58.7)	19 (41.3)	
Drink				0.071
Yes	34	15(44.12)	19(55.88)	
No	35	23(65.71)	12(34.29)	
Smoking				0.108
Yes	51	31(60.78)	20(39.22)	
No	18	7(38.89)	11(61.11)	
Muscular invasion				0.165
Yes	48	21(43.75)	27(56.25)	
No	21	13(61.9)	8 (38.1)	
TNM stage				0.018*
I–II	43	32(74.42)	11(25.58)	
III–IV	26	12(46.15)	14(53.85)	
Lymphatic metastasis				0.029*
Positive	31	22 (70.97)	9 (29.03)	
negative	38	17 (44.74)	21 (55.26)	

*Note*: *represents statistically significant.

### 3.2 CircLDLRAD3 inhibits OSCC cells proliferation, migration and invasion

**FIGURE 2:**
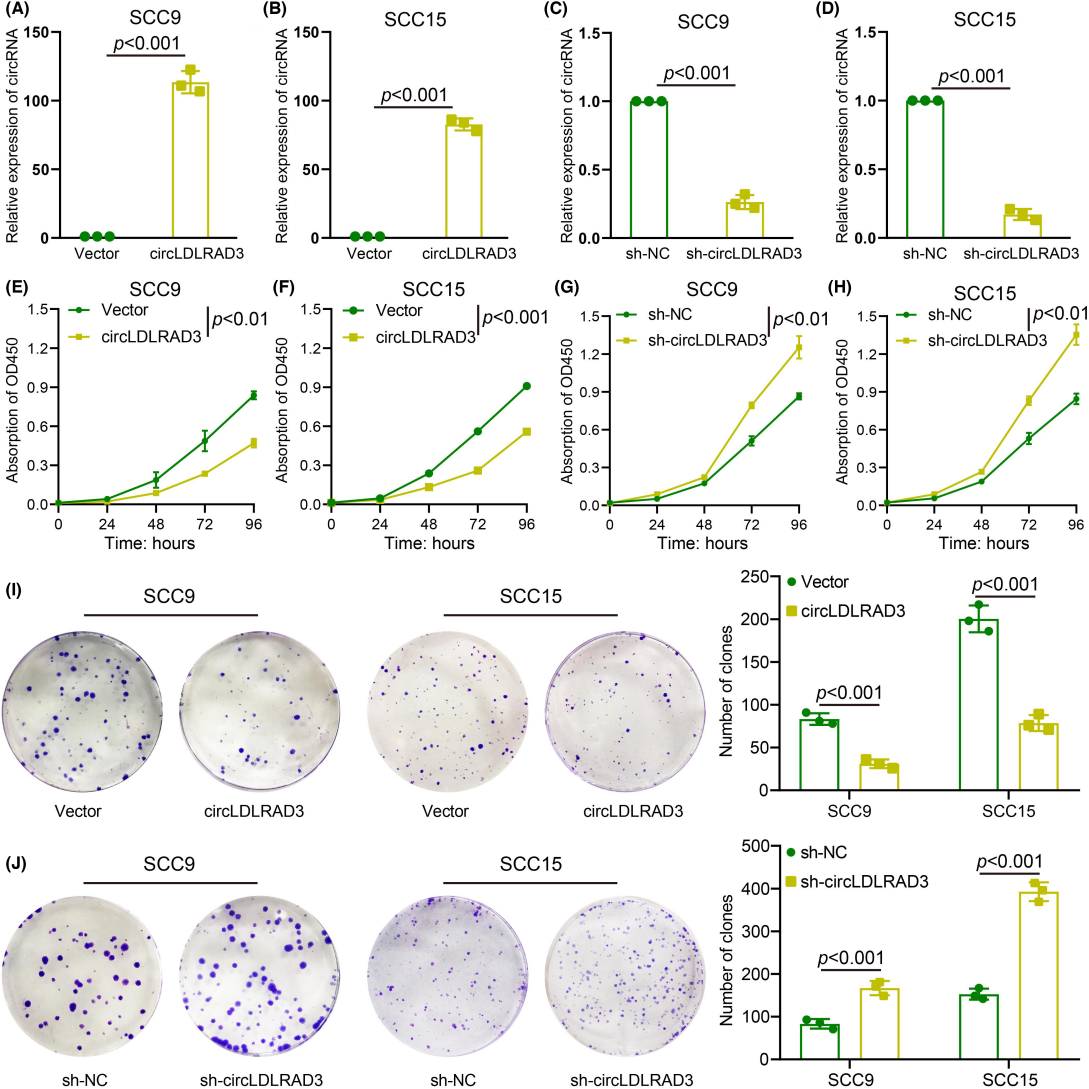
CircLDLRAD3 inhibits proliferation of OSCC cells (A, B) qRT‐PCR verified circLDLRAD3 overexpression in OSCC cells. (C, D) qRT‐PCR verified circLDLRAD3 knockdown in OSCC cells via shRNA. (E, F) circLDLRAD3 overexpression could inhibit SCC9 (E) and SCC15 (F) cell proliferation detected by CCK‐8 assay. (G, H) circLDLRAD3 knockdown could enhance SCC9 (G) and SCC15 (H) cell proliferation detected by CCK‐8 assay. (I) Colony formation assay showed circLDLRAD3 overexpression inhibit OSCC cell proliferation. (J) Colony formation assay showed circLDLRAD3 knockdown promotes OSCC cell proliferation. ** *p* < 0.01; *** *p* < 0.001.

To explore the function of circLDLRAD3 in OSCC, we constructed a circLDLRAD3 overexpression vector and knockdown shRNA specifically targeting the back splicing sites. The data of qRT‐PCR verified that circLDLRAD3 overexpression plasmids could successfully increase circRNA overexpression and circLDLRAD3 shRNA knocked down circLDLRAD3 expression in OSCC cells (Figure 2A–D). The CCK8 assay showed that overexpression of circLDLRAD3 inhibited NPC cell proliferation, and knockdown of circLDLRAD3 promoted NPC cell proliferation (Figure 2E–H). Moreover, colony formation assay further showed that circLDLRAD3 could significantly inhibit OSCC cell proliferation (Figure 2I,J).

**FIGURE 3:**
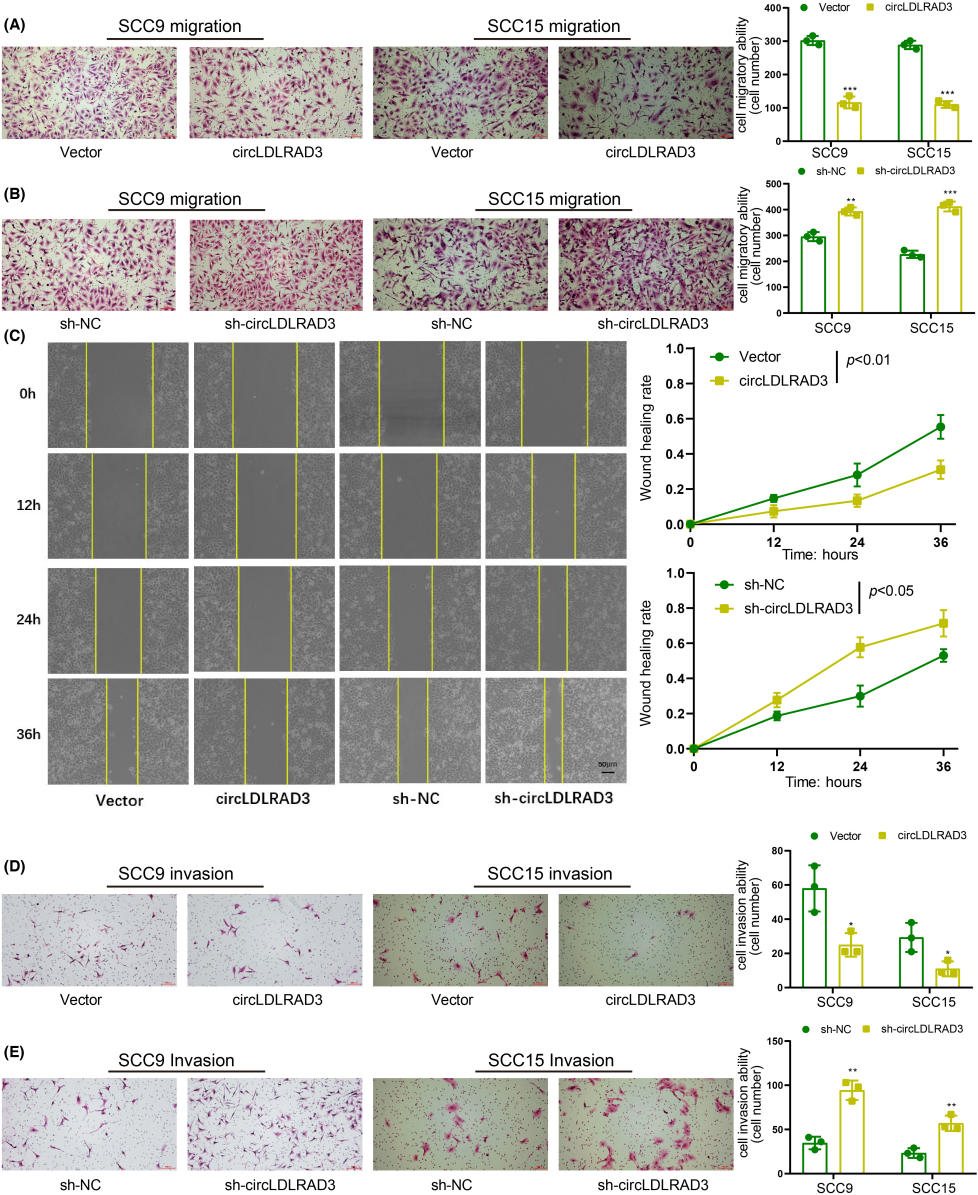
CircLDLRAD3 inhibits OSCC cells migration and invasion. (A) transwell migration assay showed circLDLRAD3 overexpression inhibit OSCC cell migration. (B) transwell migration assay showed circLDLRAD3 knockdown promotes OSCC cell migration. (C) Wound healing assay showed circLDLRAD3 inhibits OSCC cell migration. (D) transwell invasion assay showed circLDLRAD3 overexpression inhibit OSCC cell invasion. (E) transwell invasion assay showed circLDLRAD3 knockdown promotes OSCC cell invasion. Scar bar = 50 μm; ** *p* < 0.01; *** *p* < 0.001.

Subsequently, transwell migration assay was performed to explore the effect of circRNA on cell migration. Results of transwell showed that the migration capacity of OSCC cells was obviously inhibited with circLDLRAD3 overexpression and significantly enhanced upon circRNA expression inhibition (Figure 3A,B). The results of wound healing experiments showed that overexpression of circLDLRAD3 reduced OSCC cell migration and knockdown of circLDLRAD3 promote such phenotype (Figure 3C). Additionally, transwell invasion assay indicated that the invasive capacity of OSCC cells were remarkably suppressed upon ectopic expression of circLDLRAD3 but significantly increased by downregulation of circLDLRAD3 (Figure 3D,E). Taken together, these findings indicated that circLDLRAD3 inhibits OSCC cell proliferation, migration, and invasion in vitro.

### 3.3 CircLDLRAD3 inhibits OSCC tumour growth and lung metastasis in vivo

**FIGURE 4:**
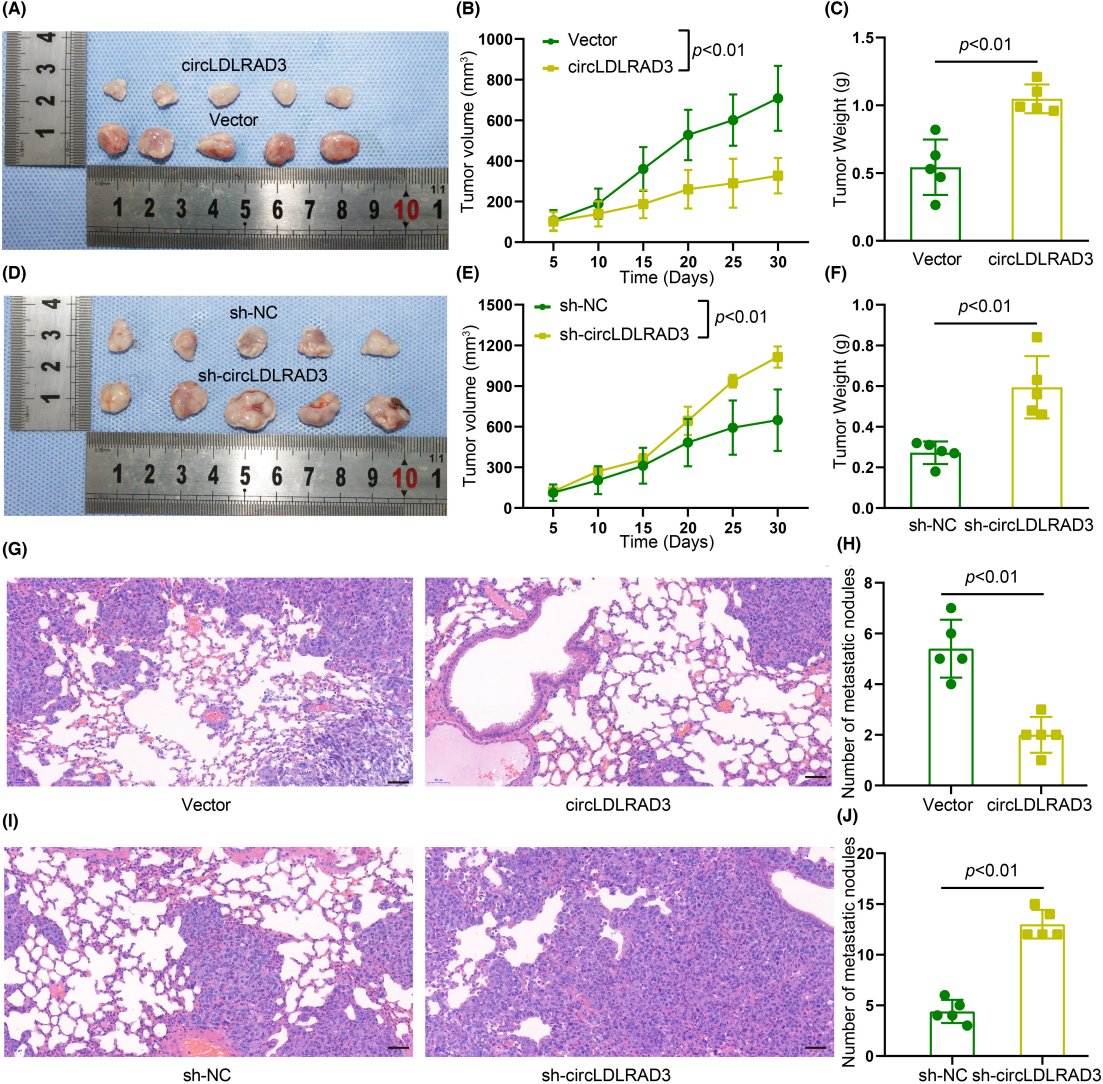
CircLDLRAD3 inhibits OSCC cell growth and metastasis in vivo. (A) Representative tumour images in circLDLRAD3 overexpression and control group. (B) Tumour volume growth curve at different time‐points. (C) Tumour weight in circLDLRAD3 overexpression and control group at the end of experiment. (D) Representative tumour images in circLDLRAD3 knockdown and control group. (E) Tumour volume growth curve at different time‐points. (F) Tumour weight in circLDLRAD3 knockdown and control group at the end of experiment. (G) Representative lung metastasis nodules in circLDLRAD3 overexpression and control group. (H) Number of metastatic nodules in circLDLRAD3 overexpression and control group. (I) Representative lung metastasis nodules in circLDLRAD3 knockdown and control group. (J) Number of metastatic nodules in circLDLRAD3 knockdown and control group. Scar bar = 50 μm; ** *p* < 0.01; *** *p* < 0.001.

As circLDLRAD3 could inhibit OSCC cell proliferation, migration, and invasion in vitro, we next explored the effects of circLDLRAD3 on OSCC tumour growth and metastasis in vivo. OSCC cells with circLDLRAD3 stably overexpressed or knockdown were inoculated into female 5‐week‐old nude mice to establish a cutaneous xenograft model. The results of subcutaneous tumorigenesis experiments showed that tumours from circLDLRAD3‐overexpressing cell were smaller, while the tumours from circLDLRAD3 knockdown group presented larger tumours than the control group (Figure 4A–F). The results of the tail vein lung metastasis assay showed that the number of metastatic nodules implanted in the lung from circLDLRAD3 overexpression group was significantly lower than that from the control group, while there was an increase in the number of metastatic nodules in the circLDLRAD3 knockdown group as compared with the control group (Figure 4G–J). Taken together, the above results suggested that circLDLRAD3 inhibits the tumour growth and lung metastasis of OSCC both in vitro and in vivo.

### 3.4 CircLDLRAD3 could directly interact with miR‐558

**FIGURE 5:**
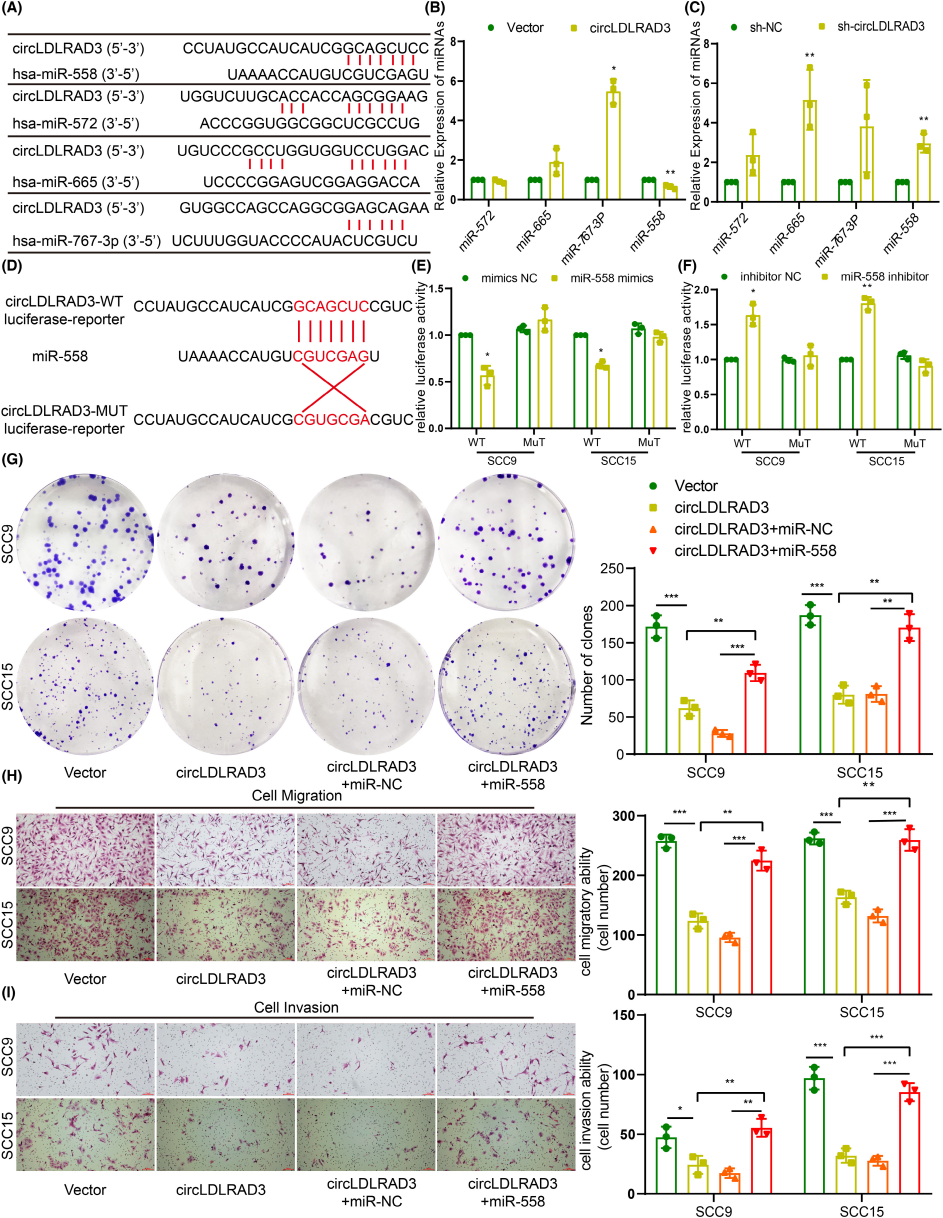
CircLDLRAD3 could directly interact with miR‐558. (A) Predicted binding sites between circLDLRAD3 and four miRNAs (miR‐572, miR‐665, miR‐767‐3p, miR‐558). (B) qRT‐PCR detection of miRNAs expression upon circLDLRAD3 overexpression. (C) qRT‐PCR detection of miRNAs expression upon circLDLRAD3 knockdown. (D) Construction of circLDLRAD3‐MUT luciferase reporter vector and circLDLRAD3‐WT luciferase reporter. (E, F) Dual luciferase assay examination of the bind between miR‐558 and circLDLRAD3‐WT luciferase reporter or circLDLRAD3‐MUT luciferase reporter. (G) Colony formation assay showed circLDLRAD3 overexpression inhibit OSCC cell proliferation, and miR‐558 reverse this effect. (H and **I**) Transwell assay showed miR‐558 could reverse the effect of circLDLRAD3 in inhibiting OSCC cell migration (E) and invasion (F). Scar bar = 50 μm; **p < 0.01; *** *p* < 0.001.

To explore the molecular mechanism underlying circLDLRAD3 in regulating OSCC growth and metastasis, we first reviewed the subcellular localization of circLDLRAD3 in OSCC. As was revealed by the FISH and nucleus‐cytoplasma separation test, the results showed that circLDLRAD3 was mainly expressed in the cytoplasma of OSCC cells (Figure 1F, G). Previous studies pointed that circRNAs in the cytoplasma are more likely to serve as a ‘molecular sponge’ for miRNA.^30^, ^31^, ^32^ Based on this speculation, we used two databases to predict potential miRNAs that could bind to circLDLRAD3, and found four miRNAs (miR‐572, miR‐665, miR‐767‐3p, miR‐558) existed in both datasets (Figure 5A). As consequence, we examined the effect of circLDLRAD3 on the expression levels of four miRNAs (miR‐572, miR‐665, miR‐767‐3p, miR‐558) via qRT‐PCR. The results showed that miR‐558 expression was significantly decreased in circLDLRAD3 overexpression cells and increased upon circLDLRAD3 knockdown, suggesting that circLDLRAD3 could negatively regulate the expression of miR‐558 (Figure 5B,C).

Subsequently, to explore if circLDLRAD3 could directly bind miR‐558, we predicted their binding sites and constructed dual‐luciferase reporter gene for binding sites between circLDLRAD3 and miR‐558 (Figure 5D). We then co‐transfected miR‐558 mimics with the circLDLRAD3‐MUT luciferase reporter vector, circLDLRAD3‐WT luciferase reporter vector into the SCC9 and SCC15 cell. The data of dual‐luciferase reporter assay demonstrated that the miR‐558 mimics strongly suppressed the activity of circLDLRAD3‐WT luciferase reporter but had no effect on the circLDLRAD3‐MUT luciferase reporter (Figure 5E,F). Together, our data suggested that circLDLRAD3 could function as a “molecular sponge” for miR‐558.

### 3.5 CircLDLRAD3 executed its tumour suppressor effect through miR‐338‐3p/Smad4 regulatory axis

As CircLDLRAD3 could directly bind miR‐558 and regulate its expression, we thus speculated whether circLDLRAD3 may execute its tumour suppressor effect through miR‐558. To confirm this speculation, we transfected miR‐558 mimics in the circLDLRAD3 overexpression OSCC cells. The results of colony formation assay showed that overexpression of miR‐558 mimics effectively rescued the effect of circLDLRAD3 on OSCC cell proliferation (Figure 5G). Similarly, miR‐558 mimics could remarkably reverse the suppressive role of circLDLRAD3 on OSCC cell migration and invasion as showed by the transwell assays (Figure 5H,I). In brief, these findings showed that circLDLRAD3, at least to some extent, executed its tumour suppressor effect on OSCC cell proliferation and migration via miR‐558.

### 3.6 Smad4 is targeted by miR‐558 and mediated the tumour suppressor role of CircLDLRAD3

**FIGURE 6:**
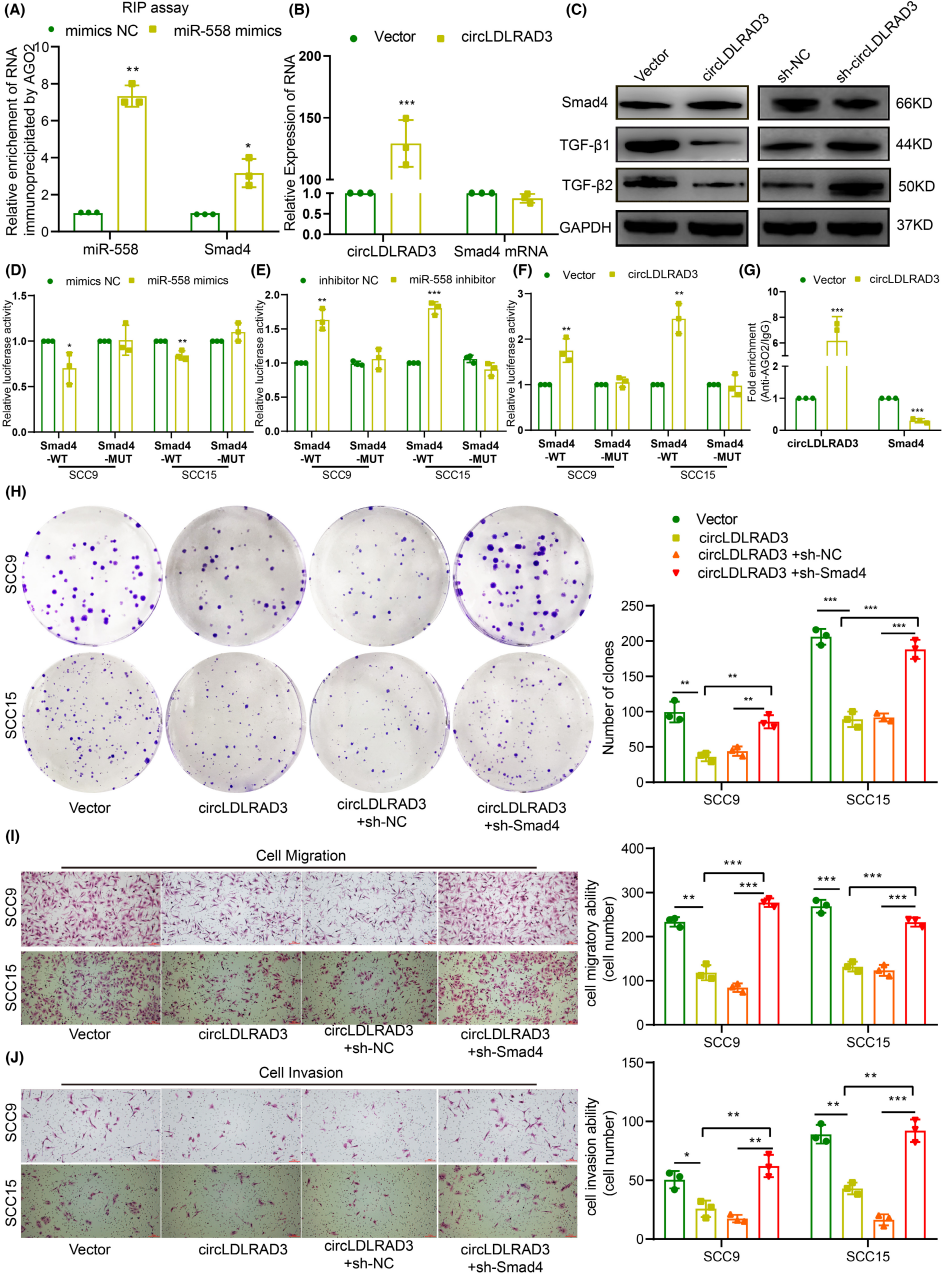
Smad4 is targeted by miR‐558 and mediated the tumour suppressor role of CircLDLRAD3. (A) RIP experiments showed miR‐558 could significantly increase the enrichment of miR‐558 and the Smad4 mRNA level in the immunoprecipitated components of Ago2 antibody. (B) qRT‐PCR detection of Smad4 expression under overexpression of circLDLRAD3. (C) Western blot detection of Smad4 expression under overexpression of circLDLRAD3. (D and E) Dual luciferase assay examination of the bind between miR‐558 and Smad4‐WT luciferase reporter or Smad4‐MUT luciferase reporter. (F) Dual luciferase assay examination of the effects of circLDLRAD3 on Smad4‐WT luciferase reporter or Smad4‐MUT luciferase reporter. (G) RIP assay showing that circLDLRAD3 can compete with the Smad4 mRNA for the binding of miR‐558. (H) Colony formation assay showed circLDLRAD3 overexpression inhibit OSCC cell proliferation, and knockdown of Smad4 reverse this effect. (I and J) Transwell assay showed knockdown of Smad4 could reverse the effect of circLDLRAD3 in inhibiting OSCC cell migration (I) and invasion (J). Scar bar = 50 μm; ** *p* < 0.01; ***p < 0.001.

The potential targets of miR‐558 were predicted by TargetScan (https://www.targetscan.org/vert_72/).^33^ According to the bioinformatic prediction and experimental results, we select Smad4 as target of miR‐558. Subsequently, we used RIP experiments to verify whether miR‐558 and Smad4 form a complex with Ago2 protein to exert their biological effects. Results of RIP experiments showed that overexpression of miR‐558 could significantly increase the enrichment of miR‐558 and the Smad4 mRNA level in the immunoprecipitated components of Ago2 antibody (Figure 6A). As circLDLRAD3 function through miR‐558, we then detect the expression of Smad4 upon circLDLRAD3 overexpression. The results showed that overexpression of circLDLRAD3 could elevate the expression of Smad4 at translational level (Figure 6B,C). Subsequently, to explore whether miR‐558 could directly bind Smad4, we constructed dual‐luciferase reporter gene for binding sites between Smad4 and miR‐558. We then co‐transfected miR‐558 mimics or miR‐558 inhibitors with the Smad4 luciferase reporter vectors into the SCC9 and SCC15 cell. The result of luciferase reporter assay showed that miR‐558 mimics suppressed the activity of Smad4‐WT luciferase reporter but had no effect on the Smad4‐MUT luciferase reporter, while miR‐558 inhibitors enhanced the activity of Smad4‐WT luciferase reporter (Figure 6D,E). Meanwhile, circLDLRAD3 could also enhance the Smad4‐WT luciferase reporter activity (Figure 6F). RIP results further showed that Overexpression of circLDLRAD3 caused a significant decrease in the enrichment of Smad4 transcripts immunoprecipitated by Ago2 (Figure 6G).

Considering the effect of miR‐558 on Smad4, we next explored whether circLDLRAD3 execute its tumour suppressor effect through Smad4. We knocked down Smad4 expression in the circLDLRAD3 overexpression OSCC cells. The results of colony formation assay showed that knockdown of Smad4 effectively rescued the effect of circLDLRAD3 on OSCC cell proliferation (Figure 6H). Similarly, transwell migration and invasion results showed that knockdown of Smad4 could remarkably reverse the suppressive role of circLDLRAD3 on OSCC cell migration and invasion (Figure 6I,J). In brief, these findings showed that circLDLRAD3 could execute its tumour suppressor effect on OSCC cell proliferation and migration via miR‐558/Smad4 axis.

### 3.7 CircLDLRAD3 regulate the TGF‐β signalling pathways through miR‐558/Smad4 axis

**FIGURE 7:**
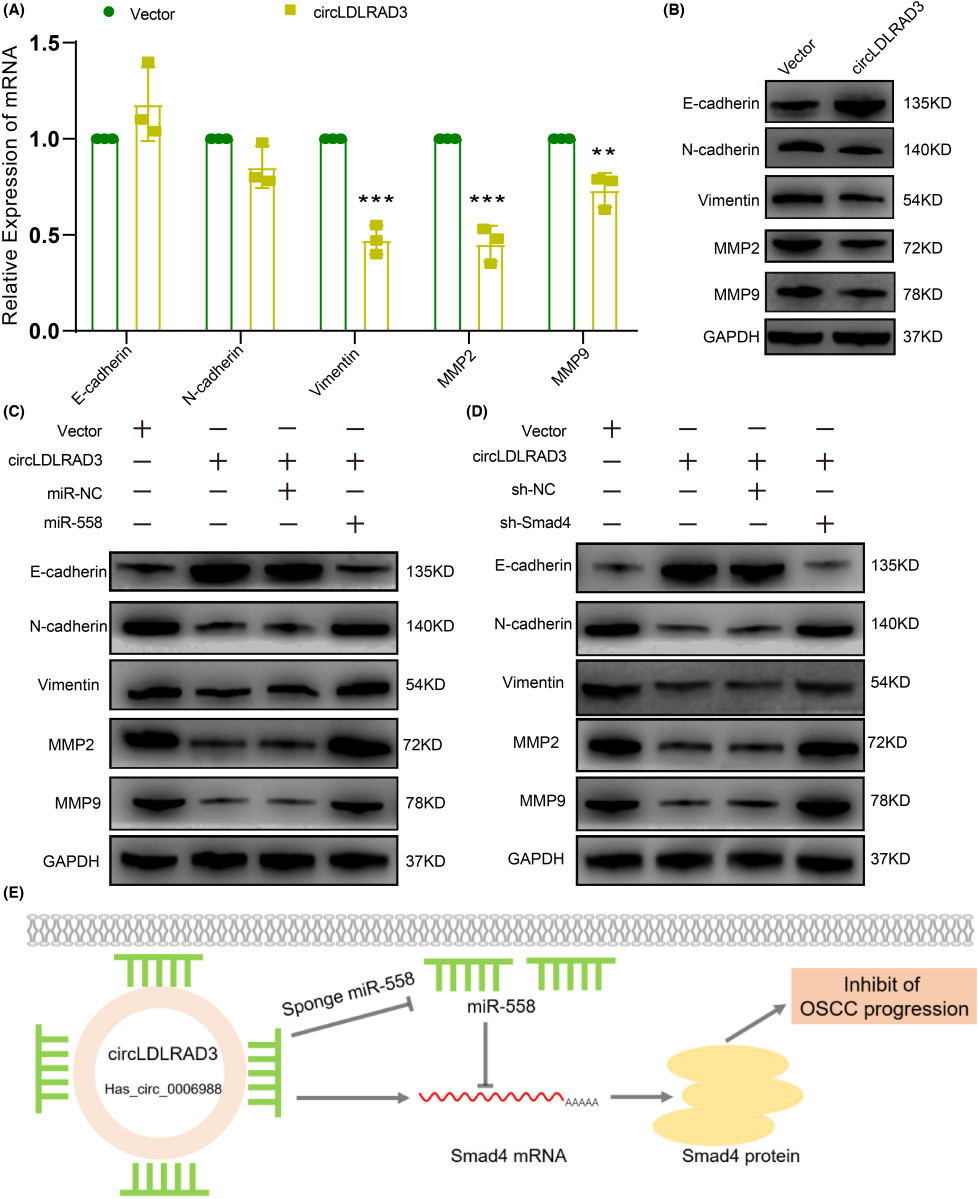
CircLDLRAD3 regulate the TGF‐β signalling pathways through miR‐558/Smad4 axis. (A) qRT‐PCR detection of EMT markers expression under overexpression of circLDLRAD3. (B) Western blot detection of EMT markers expression under overexpression of circLDLRAD3. (C) miR‐558 could reverse the effect of circLDLRAD3 in regulating EMT markers expression. (D) Knockdown of Smad4 could reverse the effect of circLDLRAD3 in regulating EMT markers expression. (E) Schematic diagram of the CircLDLRAD3 functions as a tumour‐suppressor to inhibit Oral squamous cell carcinoma progression by regulating miR‐558/Smad4 axis. **p < 0.01; *** *p* < 0.001.

Smad4 is a central signalling component of TGF‐β and deficiency of Smad4 could lead to the release of TGF‐β. Our results showed that overexpression of circLDLRAD3 could significantly increase the expression of Smad4 and thus led to a decreased expression of TGF‐β, while knockdown of circLDLRAD3 increased the expression of TGF‐β (Figure 6C). TGF‐β is an important growth factor responsible for tumour progression through promoting cancer cell proliferation and epithelial‐mesenchymal transition (EMT). Based on this, we then detect the expression of EMT markers upon circLDLRAD3 overexpression. The data showed that overexpression of circLDLRAD3 could inhibit the occurrence of EMT in OSCC cells as revealed by increased expression of epithelial marker E‐cadherin, reduced expression of the mesenchymal‐associated markers N‐cadherin, vimentin, and invasion associated markers MMP2 and MMP9 (Figure 7A,B). Meanwhile, the effect led by circLDLRAD3 overexpression could be reversed by miR‐558 mimics as well as Smad4 knockdown (Figure 7C,D). Taken together, the above results showed that CircLDLRAD3 regulate the TGF‐β signalling pathways to influence EMT through miR‐558/Smad4 axis (Figure 7E).

## 4 DISCUSSION

In recent years, it has been well documented that circRNAs can absorb miRNAs through sponge action, affecting the level of miRNAs, which in turn regulates the expression of related miRNA‐targeted genes and ultimately exerts a pro‐ or anti‐cancer effect.^30^, ^34^, ^35^, ^36^ In the present study, we confirmed that circLDLRAD3 could inhibit the malignant biological function of squamous cancer cells, and we speculated that it might act through an endogenously competitive mechanism. Through the combination of a series of bioinformatic analysis and experimental results, our study found that miR‐558 was significantly decreased after overexpression of circLDLRAD3, while increased upon circLDLRAD3 knockdown. Further results revealed that circLDLRAD3 could positively regulate Smad4 expression through sponging miR‐558, thus regulating the TGF‐β signalling pathways to influence EMT. These findings demonstrated that circLDLRAD3 played a tumour suppressor role in OSCC.

Although miR‐558 function has not been extensively studied, published findings suggest that miR‐558 has pro‐cancer effects.^37^ Upregulation of miR‐558 expression in neuroblastoma is associated with poor survival in neuroblastoma patient. MiR‐558 promotes hypoxia‐inducible factor 2α (HIF‐2α) expression through binding to target genes, thereby promoting tumorigenesis and invasiveness in neuroblastoma.^38^ Zheng et al^37^ showed that endogenous miR‐558 decreased the binding of Smad4 to HPSE, activated the transcription and expression of HPSE, and promoted tumorigenesis and invasion of gastric cancer cells both in vitro and in vivo. MiR‐558 was further demonstrated by Shohet et al^39^ to significantly increase cell proliferation, colony formation and tumour growth in vivo. They also found that miR‐558 was upregulated in lung cancer and could promote proliferation and invasion of lung cancer cells, suggesting that miR‐558 played an oncogene role. In our study, given that circLDLRAD3 could negatively regulate the expression of miR‐558 and miR‐558 played a pro‐oncogenic role in cancer, we hypothesized that circLDLRAD3 might regulate the expression of its target gene Smad4 through regulating miR‐558. We co‐transfected circLDLRAD3 and miR‐558 in squamous carcinoma cells and showed that miR‐558 could restore the effect of circLDLRAD3 on cell functions. In addition, we also verified the effect of circLDLRAD3 and miR‐558 co‐transfection on EMT‐related markers and MMP family at the translational level, and the protein expression were consistent with the results of cell function experiments. The above study confirmed the negative regulatory effect of circLDLRAD3 on miR‐558.

In our study, RT‐qPCR and western blot assays were used to examine the changes of Smad4 at transcriptional and translational levels after overexpression and knockdown of circLDLRAD3. The results showed no significant changes of Smad4 at the transcriptional level, which demonstrated that circLDLRAD3 did not affect Smad4 expression at the transcriptional level. The results also showed that overexpression of circLDLRAD3 could increase Smad4 expression while decrease TGF‐β1 and TGF‐β2 expression. Conversely, knockdown of circLDLRAD3 could result in a reduced expression of Smad4 and increased expression of TGF‐β1 and TGF‐β2, suggesting that circLDLRAD3 could modulate the expression of Smad4 and TGF. As Smad4 is a protein that transmits extracellular signals to the nucleus and is an intracellular transmitter of the TGF‐β signalling pathway. Increased Smad4 could inhibit the influence and regulation of TGF‐β on cell biological activities and plays a tumour suppressor role. These previous findings were consistent with our results that circLDLRAD3 could regulate the TGF‐β signalling pathway.

In this study, we verified the binding of miR‐558 to Smad4 using RNA‐binding protein immunoprecipitation assay, and Smad4 exerted a tumour suppressor role in cancer. We hypothesized that circLDLRAD3 may exert tumour suppressor effects by regulating the expression of miR‐558 as well as its target gene Smad4. We co‐transfected circLDLRAD3 and Smad4 in squamous carcinoma cells and showed that Smad4 could restore the effect of circLDLRAD3 on cell functions. In addition, we also verified the effect of circLDLRAD3 and Smad4 co‐transfection on EMT‐related markers and MMP family at translational level, and the results showed that Smad4 could restore the effect of circLDLRAD3 on EMT‐related markers and MMP family at translational level.

In conclusion, our data first discovered circLDLRAD3 is downregulated in OSCC and verified its tumour suppressor function and mechanism in OSCC through sponging miR‐558 to regulate miR‐558/Smad4/TGF‐β axis. The characterization of such regulating network uncovers an important mechanism underlying OSCC progression, which could provide promising targets for targeted therapy strategies for OSCC in the future.

## AUTHOR CONTRIBUTIONS

**Xue Zhang:** Conceptualization (equal); data curation (equal); writing – original draft (equal); writing – review and editing (equal). **Guang‐Yu Guo:** Data curation (equal); formal analysis (equal); investigation (equal). **Ru‐Yue Liu:** Data curation (equal); investigation (equal); methodology (equal). **Ting Wu:** Formal analysis (equal); investigation (equal); writing – review and editing (equal). **Zhen‐Hua Wang:** Conceptualization (equal); data curation (equal); formal analysis (equal); supervision (equal); validation (equal); writing – review and editing (equal). **Zhong‐Ti Zhang:** Conceptualization (equal); data curation (equal); supervision (equal); visualization (equal); writing – original draft (equal); writing – review and editing (equal).

## FUNDING INFORMATION

The present study was supported by the Liaoning Province Central Government Guiding Local Science and Technology Development Funds (2022JH6/100100058).

## CONFLICT OF INTEREST STATEMENT

These authors declared no competing interests in this research.

## Supporting information


**Table S1.** The antibodies used in this study.

## Data Availability

All data generated or analyzed during this study are included in this published article and its supplementary information files.
