# Supplementary material for: CircLDLRAD3 inhibits Oral squamous cell carcinoma progression by regulating miR‐558/Smad4/TGF‐β
Source: J Cell Mol Med. 2023 Aug 10;27(21):3271–85. doi: 10.1111/jcmm.17898 (PMC10623532; doi:10.1111/jcmm.17898)
Supplement: Supplementary file 1 — Table S1. The antibodies used in this study. [file JCMM-27-3271-s001.docx]

Table S1 The antibodies used in this study.

| Antibodies | Product company |
| --- | --- |
| Anti-Smad4 | Bioss, Beijing, China |
| Anti-TGFβ1 | Bioss, Beijing, China |
| Anti-TGFβ2 | Bioss, Beijing, China |
| Anti-MMP9 | Bioss, Beijing, China |
| Anti-MMP2 | Bioss, Beijing, China |
| Anti-GAPDH | Bioss, Beijing, China |
| Anti-βcatenin | Bioss, Beijing, China |
| Anti-E-cadherin | Proteintech, Wuhan, China |
| Anti-N-cadherin | Proteintech, Wuhan, China |
| Anti-Vimentin | Proteintech, Wuhan, China |
| Anti-AGO2 | Abcam, USA |
